# Analysis of the Evolution Game of Construction and Demolition Waste Recycling Behavior Based on Prospect Theory under Environmental Regulation

**DOI:** 10.3390/ijerph15071518

**Published:** 2018-07-18

**Authors:** Hong Shen, Ying Peng, Chunxiang Guo

**Affiliations:** 1 College of Architecture & Environment, Sichuan University, No. 24 South Section 1, Yihuan Road, Chengdu 610065, China; 2017223050039@stu.scu.edu.cn (H.S.); pengying@scu.edu.cn (Y.P.); 2 College of Business, Sichuan University, No. 24 South Section 1, Yihuan Road, Chengdu 610065, China

**Keywords:** construction and demolition waste recycling, environmental regulation, evolutionary game theory, prospect theory

## Abstract

With the development of the construction industry, increasing concern over construction and demolition waste (CDW) has initiated a wave of environmental regulation by the government in order to reduce the environmental impact and ensure sustainable development. Research on behavioral decision-making can offer a theoretical basis for the government and individuals. This paper aims to study the behavioral decision-making of stakeholders in CDW recycling under environmental regulation. Considering the limited rationality of stakeholders and the difference in reference points, an evolutionary game model including contractors and manufacturers of construction materials is proposed based on the prospect theory of behavioral economics. The results indicate that, only when the perceived benefits of one or both stakeholders for participation under the environmental regulation exceed those for non-participation, can the CDW recycling system eventually evolve to a stable state in which both stakeholders choose to participate. In addition, factors such as the initial strategy, production cost, technology, subsidies, recycling benefits, and the degree of perception of the stakeholders, exert certain influences on the stable state. To attain the required stable state, the government should increase the subsidies for the stakeholders and strengthen the publicity regarding recycling effects to improve the perceived benefits.

## 1. Introduction

With the rapid development of China’s economy and urbanization, the construction industry has enjoyed continuous development and has gradually become one of the pillar industries of the national economy. It is closely related to the economic development of the entire country and the improvement in people’s lives. While the construction industry has made tremendous contributions to the development of the entire society, the amount of construction and demolition waste (CDW) has also grown rapidly, accounting for about 40% of urban waste in China.

Relevant studies [1,2,3] have shown that the recycling of CDW can efficiently save resources, reduce pollution, stimulate the economy, and obtain greater social, economic, and environmental effects. Japan has enacted a lot of laws to promote the recycling of CDW, such as the ‘Recycle Act’ and ‘Act on recycling construction related materials’, which require the government projects to use recycled construction materials and offer certain subsidies for recycling enterprises [4]. China has issued ‘the interim measures on the administration of financial subsidies for recycling construction materials’, which stipulates the subsidies for re-manufacturers of construction materials, to improve the level of recycling of CDW [5]. However, compared with Japan, Korea, Germany, and other countries where the recycling rate of CDW is more than 90%, the recycling rate of CDW in China is less than 5% [6]. On the one hand, the vast majority of CDW in China is usually simply disposed of by open storage or landfill, and illegal dumping by contractors occurs frequently. On the other hand, re-manufacturers of construction materials lack recycled materials; therefore, it is difficult for the CDW recycling industry to operate on CDW recycling. As a result, resource shortage, environmental pollution, and other problems occur one after another. Given that the situation for CDW is not optimistic, the government has paid special attention to CDW recycling management and has adopted corresponding regulatory measures, such as penalties for violations and supporting policies for the recycling of CDW. How to promote the development of the CDW recycling industry has become the focus of the government, the public, and the industry.

In recent years, an increased number of studies have been published in academic literature about the recycling of CDW focusing on various aspects. Management of CDW is one of the most important directions. For example, Gluzhge [7] first proposed the concept for the recycling of CDW and ensured its positive contribution compared with traditional disposal methods. Shen et al. [8] divided the research of CDW management (CDWM) into three areas: waste classification, waste management strategy, and waste disposal technology. In addition, CDW management strategies also include avoiding, reducing, reusing, and recycling waste. Yuan et al. [9] summarized the latest research trends in CDWM by analyzing eight major international journal publications from 2000 to 2009, and found that surveys and case studies are the main methods for the data collection of CDW.

Several papers have evaluated the effects of the recycling of CDW. Mohamed et al. [10] established a quantitative model of environmental and economic benefits for three disposal methods of CDW, and found that the most environmentally friendly method is recycling, followed by incineration, and the last is landfilling. Ibrahim [11] selected waste management data of the actual construction projects in University of Massachusetts Amherst. By analyzing the actual case data, he proposed a derived statistical model to provide a basis for CDW quantification, recycling costs, and overall effects in order to improve the recycling rate of CDW.

In addition, the identification of factors that influence CDWM is imperative towards improving waste recycling practice. As mentioned by Lu et al. [12], seven critical success factors that influence the effective management of CDW are determined through conducting semi-structured interviews with construction workers and government officials. Management regulation is the most important factor. Yuan et al. [13] studied the impact of different CDW disposal fees on the results based on system dynamics theory in order to promote the recycling of CDW. Along the same lines, Wang et al. [14] explored the influence of designers’ decisions and behavior on CDWM. The results indicate that the use of prefabricated components exerts the largest influence on the design of construction waste reduction, followed by few design modifications and waste reduction investment. Moreover, according to the questionnaire survey of Jin et al. [15], we can know that China is still at an early stage in the recycling of CDW and government supervision is an important factor in its development. At the same time, economic feasibility is the leading factor.

It is becoming increasingly clear that government intervention in the form of environmental regulation for CDW is potentially beneficial. For example, Liu et al. [16], from the perspective of the contractors and society, simulated the impact of positive and negative economic measures, including subsidies and fines, on the CDW disposal costs and effects. In the context of Brazil’s regulation, which mandated waste to contribute to the reverse supply chain, Ghisolfi et al. [17] measured the influence of legal incentives and recycler abilities on the reverse supply chain, and found that the current development of waste recycling still requires legal incentives.

In summary, most of the above conducted qualitative and quantitative research studies were based on the methods of questionnaires, case studies, numerical analysis, system simulations, etc., with more attention paid to the external structure of the CDW recycling industry. Nevertheless, research on the internal mechanism of the behavioral decision-making of CDW recycling is an effective way to increase the level of recycling and optimize the allocation of resources under the background of information asymmetry and incompleteness. With the development of game theory, evolutionary games have provided a practical method for studying behavioral decisions. To study the interactions among rational players whose strategic behaviors are influenced by each other, the studies of Maynard Smith and Price [18], Taylor and Jonker [19], Friedman [20], and Weibull [21] make evolutionary game theory increasingly mature.

There are few studies on behavioral decision-making related to the recycling of CDW, and internal mechanism research is still limited. Only Liu et al. [22] analyzed construction waste disposal enterprises and building materials production enterprises by an evolutionary game based on the theory of circular economy. Their study shows that game players would not like to cooperate because of the incremental cost and raw material pricing, and the government behaviors can promote the formation of a green building material industry chain. In the same lines, taking the contractor into consideration, Yuan et al. [23] explored the formation and evolutionary path of behavioral decision-making between contractors and building materials production enterprises by applying evolutionary game theory. However, both of them ignored the influence of the initial strategy and lacked numerical simulation.

In addition, although compared with the traditional classical game, the evolutionary game considers the bounded rationality of the decision makers and is thus more realistic, it still constructs the payoff matrix objectively and conducts analysis based on the classical expected utility theory [24]. It does not completely conform to the bounded rationality hypothesis [25] and fails to take into account the fact that the subject is susceptible to psychological factors [26], ignoring the subject’s value perception which may deviate from the actual results, such as the famous Allais paradox [27], Ellsberg Paradox [28], Asian Disease Effect [29] etc.

Based on the discussion above, in order to improve the credibility of the traditional evolutionary game and the effectiveness of the interpretation of reality, this paper attempts to introduce the prospect theory of behavioral economics into the process of evolutionary game analysis. Prospect theory was introduced in 1979 [30] by Daniel Kahneman and Amos Tversky as behavior economic theory, which describes the way in which people choose between probabilistic alternatives that involve risk when the probabilities of outcomes are known. According to the theory, people make decisions based on the potential value of losses and gains rather than the final outcome.

On the basis of overcoming the limitations of the expected utility theory, we considered the limited rationality of behavioral decision-making and the differences of reference points and proposed an internal mechanism model of CDW recycling under environmental regulation. The bounded rationality assumption was applied to the perception and decision-making process of contractors and manufacturers of construction materials to study the behavioral decision-making evolutionary path, stable strategies, and the impact of the factors, so as to provide constructive suggestions for the government to formulate environmental regulatory policies.

The remainder of current study is organized as follows. In Section 2, the assumptions are explained and the evolution game model between contractors and manufacturers of construction materials is described. Section 3 focuses on the equilibrium analysis of the evolutionary game and the impact of factors on the stable strategies. In Section 4, a numerical simulation is presented based on the model in Section 2. Finally, Section 5 draws conclusions and further research directions.

## 2. Model and Assumptions

**Figure 1:**
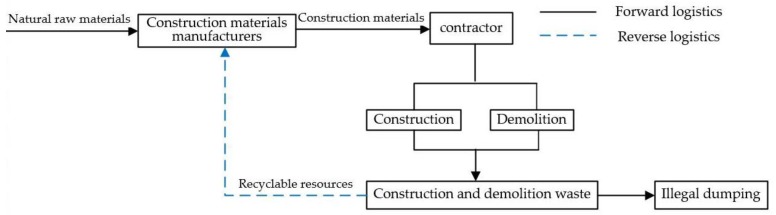
The CDW recycling supply chain.

In reality, the difference between re-manufacturers of construction materials and traditional manufacturers of construction materials is related to the technologies used, the sources of material, the future markets, and the profit. However, this study mainly considers the difference in production materials, costs, and technology between them, and assumes that their products are homogeneous. The former production materials are CDW, and the latter are natural raw materials. Therefore, this study abstracts both of them as construction materials manufacturers (manufacturers). On top of that, in order to facilitate the analysis, this study links the contractors to manufacturers directly instead of employing the CDW recycling center as a bridge, as shown in Figure 1.

In fact, manufacturers and contractors are not the only stakeholders. However, these are the stakeholders considered in this study. This study examines the behavioral decision-making of stakeholders of the CDW recycling industry under environmental regulation, i.e., whether the manufacturers choose recycling production or whether the contractors choose to participate in the recycling of CDW. It analyzes the formation and evolutionary path of the behavioral decision-making and the impact of factors among stakeholders in the CDW recycling industry under environmental regulation based on the model, and then provides a theoretical basis for the formulation of regulatory policies.

Considering what has been mentioned above, we propose the following assumptions:

**Assumption** **1.** In the CDW recycling supply chain, there are only two game players: the contractors and the manufacturers. The contractors face two choices: sorting and transporting the CDW to manufacturers (“participation”) and illegal dumping at the risk of discovery by the government (“non-participation”). The strategies that can be selected by the manufacturers include participation and non-participation. In the case of “participation”, the manufacturers choose CDW as production materials, which they will sort, dispose of, and use to produce the construction materials in the factory. In the case of “non-participation”, the manufacturers select natural raw materials as production materials.

**Assumption** **2.** When both stakeholders take part in the strategy of participation, the CDW recycling industry runs well and brings certain environmental and social benefits (F), and the internalization degree (i.e., the proportion of benefits that the participant can obtain from F) of the manufacturers and the contractors to these effects is β and γ, respectively. When only one or both stakeholders do not participate, there will be no environmental and social benefits.

**Assumption** **3.** If the manufacturers choose to participate, they will need to introduce equipment, technology, and personnel to sort and dispose of CDW from the contractors. This results in the recycling cost C_1_. The production cost of the finished products is C_2_, including the cost of production materials and producing. The environmental regulation subsidy from the government is K_1_C_1_; if not, the traditional production cost of the production of equal quantities of finished products is αC_2_. α is the production cost proportional coefficient between traditional production and recycling production, and it is positively correlated with the recycling rate of CDW. Regardless of which material is selected as the production material to produce the finished products, the selling price is the same and the income is R.

**Assumption** **4.** If the contractors choose to participate, they will need to spend C_3_ sorting on site and obtain environmental regulation subsidy of K_2_C_3_ from government; if not, θ is the probability of discovering the illegal dumping, which is proportional to the degree of implementation of environmental efforts by the government and T is the fine of illegal dumping.

**Assumption** **5.** *The two game players are bounded rational people. Therefore, both players make decisions based on their own perception of strategic values under the principle of maximizing profits. There is no difference between the perceived value and the actual utility of the losses and benefits determined by the two stakeholders. Only when the two stakeholders are uncertain about the costs and benefits, *etc.*, can they have the perceived utility. This perception is not a real utility situation. Its characteristics are in line with prospect theory and the perceived utility is V(Δw), whose formula is as follows* [30]:

(1) $$V(\Delta \omega )=\left\{ \begin{array}{ll} \;\Delta \omega^{a}, & \Delta \omega \geq 0\\ -\lambda {\left(-\Delta \omega \right)}^{b}, & \Delta \omega <0 \end{array}\right.$$

Here, Δω represents the difference between the actual gains and losses of the participants and the reference point, and a and b represent the degree of marginal decline of the value of the “gain” and “loss” values perceived by the decision-makers. The larger the value, the greater the degree of marginal decline (0 ≤ a,b ≤ 1); *λ* is the loss avoidance coefficient, and the larger the value, the higher the sensitivity of the decision-makers to the loss. For simplifying the analysis, this study sets the value of the reference point in the value function to zero. The perceived utility of the contractors is $V_{1}(\Delta w)$. The corresponding parameters are a_1_, b_1_, and *λ*_1_. The perceived utility of the manufacturers is $V_{2}(\Delta w)$, and the corresponding parameters are a_2_, b_2_, and *λ*_2_.

Based on the above mentioned five assumptions, the perceived payoff matrix between the contractors and the manufacturers under the environmental regulation in the CDW recycling industry is established, as shown in Table 1.

## 3. Equilibrium Analysis of Evolutionary Game

### 3.1. The Perceived Benefit of Each Game Player

In the initial stage, we assume that the proportion of the contractors that choose participation is *x*, and the proportion of the contractors that choose non-participation is 1 − *x*. Also suppose that the proportion of the manufacturers that choose participation is *y*, and the proportion of the manufacturers that choose non-participation is 1 − *y*. Obviously, 0 ≤ *x* ≤ 1, 0 ≤ *y* ≤1.

According to Table 1, the perceived benefit of the contractors for the participation strategy is:

(2) $$U_{11}=y\cdot \left[V_{1}(\gamma F)+V_{1}{(K}_{2}C_{3})-C_{3}\right]+(1-y)\cdot \left[V_{1}(K_{2}C_{3})-C_{3}\right]\;=y\cdot V_{1}(\gamma F)+V_{1}(K_{2}C_{3})-C_{3}\;$$

The perceived benefit of the contractors for the non-participation strategy is:

(3) $$U_{12}=y\cdot V_{1}(-\theta T)+(1-y)\cdot V_{1}(-\theta T)=V_{1}(-\theta T)\;$$

The average perceived benefit of the contractors is:

(4) $$\bar{U_{1}}=x\cdot U_{11}+(1-x)\cdot U_{12}=xy\cdot V_{1}(\gamma F)+x\cdot V_{1}(K_{2}C_{3})-x\cdot C_{3}+(1-x)\cdot V_{1}(-\theta T)\;$$

The perceived benefit of the manufacturers for the participation strategy is:

(5) $$U_{21}=x\cdot \left[R-C_{1}-C_{2}-V_{2}(\beta F)+V_{2}{(K}_{1}C_{1})\right]+(1-x)\cdot \left[V_{2}(K_{1}C_{1})-C_{1}-C_{2}\right]=x\cdot R+x\cdot V_{2}(\beta F)+V_{2}(K_{1}C_{1})-C_{1}-C_{2}\;$$

The perceived benefit of the manufacturers for the non-participation strategy is:

(6) $$U_{22}=x\cdot (R-\alpha C_{2})+(1-x)\cdot (R-\alpha C_{2})=(R-\alpha C_{2})\;$$

The average perceived benefit of the manufacturers is:

(7) $$\bar{U_{2}}=y\cdot U_{21}+(1-y)\cdot U_{22}=(1+xy-y)\cdot R+xy\cdot V_{2}(\beta F)+y\cdot V_{2}(K_{1}C_{1})-yC_{1}-(y-\alpha y+\alpha )C_{2}\;$$

### 3.2. The Replication Dynamic Analysis of Each Game Player

Using the asymmetric replication dynamic evolution approach [19], the replication dynamic equations of the proportion x for the contractors and the proportion y for the manufacturers are:

(8) $$\frac{dx}{\mathrm{dt}}=x\cdot (U_{11}-\bar{U_{1}})=x\cdot (1-x)\cdot (U_{11}-U_{12})=x\cdot (1-x)\cdot \left[y\cdot V_{1}(\gamma F)+V_{1}(K_{2}C_{3})-C_{3}-V_{1}(-\theta T)\right]\;$$

(9) $$\frac{dy}{\mathrm{dt}}=y\cdot (U_{11}-\bar{U_{1}})=y\cdot (1-y)\cdot (U_{21}-U_{22})=y\cdot (1-y)\cdot \left[x\cdot (R+V_{2}(\beta F))+V_{2}(K_{1}C_{1})-R-C_{1}-(1-\alpha )\cdot C_{2}\right]\;$$

Equation (8) indicates that only when *x* = 0, 1 or $y^{*}=\frac{V_{1}(-\theta T)-\left[V_{1}(K_{2}C_{3})-C_{3}\right]}{V_{1}(\gamma F)}$ does the participation strategy of the contractors reach a local stable; Equation (9) indicates that the strategy of the manufacturers to choose to participate is only locally stable when *y* = 0, 1 or $x^{*}=\frac{R-\alpha C_{2}-\left[V_{2}(K_{1}C_{1})-C_{1}-C_{2}\right]}{R+V_{2}(\beta F)}$. Therefore, the system composed of Equations (8) and (9) has equilibrium points E_1_ (0,0), E_2_ (1,0), E_3_ (0,1), and E_4_ (1,1), and when $\left\{ \begin{array}{l} V_{2}(K_{1}C_{1})-C_{1}-C_{2}<R-\alpha C_{2}<R+V_{2}(\beta F)+V_{2}(K_{1}C_{1})-C_{1}-C_{2}\\ V_{1}(K_{2}C_{3})-C_{3}<V_{1}(-\theta T)<V_{1}(\gamma F)+V_{1}(K_{2}C_{3})-C_{3} \end{array}\right.$ is satisfied at the same time, there is an equilibrium point E_5_ (x*,y*). Among them, E_1_, E_2_, E_3_, and E_4_ are the pure strategy Nash equilibrium, and E_5_ is the mixed strategy Nash equilibrium.

### 3.3. The Stability Analysis of Equilibrium Strategy

According to the method proposed by Friedman [20], the evolutionary stable strategy (ESS) of the differential equation system can be obtained from the local stability analysis of the Jacobian matrix J of the system, namely that if and only if Determinant J (Det J) > 0 and Trace J (Tr J) < 0, the point has local stability. Equations (8) and (9) constitute the system of equations, whose Jacobian matrix is:

$$J=\left[\begin{array}{cc} (1-2x)\cdot \left[y\cdot V_{1}(\gamma F)+V_{1}(K_{2}C_{3})-C_{3}-V_{1}(-\theta T)\right] & x(1-x)\cdot V_{1}(\gamma F)\\ y(1-y)\cdot \left[R+V_{2}(\beta F)\right] & (1-2y)\cdot \left[x\cdot (R+V_{2}(\beta F))+V_{2}(K_{1}C_{1})-C_{1}-(1-\alpha )C_{2}-R\right] \end{array}\right]\;$$

Det J and Tr J calculation formulas for each equilibrium point are shown in Table 2 and Table 3. We analyze various equilibrium scenarios below.

Scenario 1: When $R-\alpha C_{2}<(K_{1}C_{1}{)}^{a_{2}}-C_{1}-C_{2}$ and $-\lambda_{1}{\left(\theta T\right)}^{b_{1}}<(K_{2}C_{3}{)}^{a_{1}}-C_{3}$ are satisfied, the evolutionary stable strategy (ESS) of the system is E_4_ (1,1). That is, regardless of the contractors’ strategy, the perceived benefit of the manufacturers of the participation strategy is greater than the perceived benefit of the non-participation strategy; no matter what the strategy of the manufacturers is, the perceived benefit of the contractors choosing to participate is greater than the perceived benefit of non-participation. Thus, the two game players are more inclined to the participation strategy.

Scenario 2: When $R-\alpha C_{2}<(K_{1}C_{1}{)}^{a_{2}}-C_{1}-C_{2}$ and $(\gamma F{)}^{a_{1}}+(K_{2}C_{3}{)}^{a_{1}}-C_{3}<-\lambda_{1}{\left(\theta T\right)}^{b_{1}}$ are satisfied, that is to say, whatever the strategy of the contractors is, the perceived benefit of the manufacturers of the participation strategy is greater than the perceived benefit of the non-participation strategy; regardless of the manufacturers’ strategy, the perceived benefit of the contractors choosing to participate is less than the perceived benefit of non-participation. Thus, the evolutionary stable strategy (ESS) of the system is E_3_ (0,1).

Scenario 3: When $R+{\left(\beta F\right)}^{a_{2}}+(K_{1}C_{1}{)}^{a_{2}}-C_{1}-C_{2}<R-\alpha C_{2}$ and $-\lambda_{1}{\left(\theta T\right)}^{b_{1}}<(K_{2}C_{3}{)}^{a_{1}}-C_{3}$, the evolutionary stable strategy (ESS) of the system is E_2_ (1,0). That is, regardless of the contractors’ strategy, the perceived benefit of the manufacturers of the participation strategy is less than the perceived benefit of the non-participation strategy; whatever the strategy of manufacturers is, the perceived benefit of the contractors choosing the participation strategy is greater than the perceived benefit of non-participation.

Scenario 4: When $R+{\left(\beta F\right)}^{a_{2}}+(K_{1}C_{1}{)}^{a_{2}}-C_{1}-C_{2}<R-\alpha C_{2}$ and $(\gamma F{)}^{a_{1}}+(K_{2}C_{3}{)}^{a_{1}}-C_{3}<-\lambda_{1}{\left(\theta T\right)}^{b_{1}}$, the evolutionary stable strategy (ESS) of the system is E_1_ (0,0), that is to say, whatever the strategy of the contractors is, the perceived benefit of the manufacturers of the participation strategy is less than the perceived benefit of the non-participation strategy; regardless of the manufacturers’ strategy, the perceived benefit of the contractors choosing to participate is less than the perceived benefit of non-participation. As a result, the two game players are more inclined to the non-participation strategy.

Scenario 5: The evolutionary stable strategy (ESS) of the system is E_1_ (0,0), when $(\beta F{)}^{a_{2}}+(K_{1}C_{1}{)}^{a_{2}}-C_{1}-C_{2}<R-\alpha C_{2}$ and $(K_{2}C_{3}{)}^{a_{1}}-C_{3}<-\lambda_{1}{\left(\theta T\right)}^{b_{1}}<(\gamma F{)}^{a_{1}}+(K_{2}C_{3}{)}^{a_{1}}-C_{3}$. That is, regardless of the contractors’ strategy, the perceived benefit of the manufacturers of the non-participation strategy is greater than the perceived benefit of the participation strategy; however, the perceived benefit of the contractors is affected by the manufacturers. The contractors’ perceived benefit of non-participation is greater than the perceived benefit of participation when the manufacturers choose non-participation, but it is less than the perceived benefit when the contractors choose to participate in the case of the manufacturers choosing participation. Thus, the two game players are more inclined to the non-participation strategy.

Scenario 6: The evolutionary stable strategy (ESS) of the system is E_4_ (1,1), when $R-\alpha C_{2}<(K_{1}C_{1}{)}^{a_{2}}-C_{1}-C_{2}$ and $(K_{2}C_{3}{)}^{a_{1}}-C_{3}<-\lambda_{1}{\left(\theta T\right)}^{b_{1}}<(\gamma F{)}^{a_{1}}+(K_{2}C_{3}{)}^{a_{1}}-C_{3}$. That is to say, no matter what the strategy of the contractors is, the perceived benefit of the manufacturers of the participation strategy is greater than the perceived benefit of the non-participation strategy; however, the perceived benefit of the contractors is affected by the manufacturers. The perceived benefit of the contractors of non-participation is greater than the perceived benefit of participation when the manufacturers choose non-participation, but it is less than the perceived benefit when the contractors choose to participate in the case of the manufacturers choosing participation. Therefore, both game players tend to participate.

Scenario 7: The evolutionary stable strategy (ESS) of the system is E_4_ (1,1), when the factors satisfied ${\left(K_{1}C_{1}\right)}^{a_{2}}-C_{1}-C_{2}<R-\alpha C_{2}<R+{\left(\beta F\right)}^{a_{2}}+(K_{1}C_{1}{)}^{a_{2}}-C_{1}-C_{2}$ and $-\lambda_{1}{\left(\theta T\right)}^{b_{1}}<{\left(K_{2}C_{3}\right)}^{a_{1}}-C_{3}$. That is, regardless of the manufacturers’ strategy, the perceived benefit of the contractors for participation is greater than the perceived benefit of non-participation. However, the perceived benefit of the manufacturers is affected by the contractors. The perceived benefit of the manufacturers of non-participation is greater than the perceived benefit of participation when the contractors choose non-participation, but it is less than the perceived benefit when the manufacturers choose to participate in the case of the contractors choosing participation. As a result, the two game players are more inclined to the participation strategy.

Scenario 8: The evolutionary stable strategy (ESS) of the system is E_1_ (0,0), when ${\left(K_{1}C_{1}\right)}^{a_{2}}-C_{1}-C_{2}<R-\alpha C_{2}<R+{\left(\beta F\right)}^{a_{2}}+{\left(K_{1}C_{1}\right)}^{a_{2}}-C_{1}-C_{2}$ and $(\gamma F{)}^{a_{1}}+(K_{2}C_{3}{)}^{a_{1}}-C_{3}<-\lambda_{1}{\left(\theta T\right)}^{b_{1}}$. That is, regardless of the manufacturers’ strategy, the perceived benefit of the contractors for participation is less than the perceived benefit of non-participation. However, the perceived benefit of the manufacturers is affected by the contractors. The manufacturers’ perceived benefit of non-participation is greater than the perceived benefit of participation when the contractors choose non-participation, but it is less than the perceived benefit when the manufacturers choose to participate in the case of the contractors choosing participation. Therefore, both game players tend not to participate.

**Figure 2:**
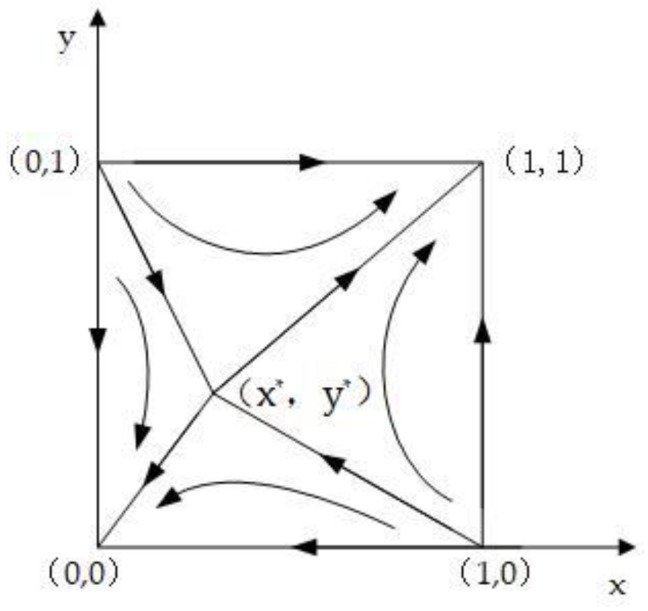
Phase diagram of the evolutionary game.

Scenario 9: The strategy of both game players is affected by the strategy of the other players, when the factors satisfy $(K_{1}C_{1}{)}^{a_{2}}-C_{1}-C_{2}<R-\alpha C_{2}<R+{\left(\beta F\right)}^{a_{2}}+(K_{1}C_{1}{)}^{a_{2}}-C_{1}-C_{2}$ and $(K_{2}C_{3}{)}^{a_{1}}-C_{3}<-\lambda_{1}{\left(\theta T\right)}^{b_{1}}<(\gamma F{)}^{a_{1}}+(K_{2}C_{3}{)}^{a_{1}}-C_{3}$. The perceived benefit of the manufacturers of non-participation is greater than the perceived benefit of participation when the contractors choose non-participation, but it is less than the perceived benefit when the manufacturers choose to participate in the case of the contractors choosing participation. Additionally, the perceived benefit of the contractors of non-participation is greater than the perceived benefit of participation when the manufacturers choose non-participation, but it is less than the perceived benefit when the contractors choose to participate in the case of the manufacturers choosing participation. Therefore, the result of the evolution of the long-term game between the two players is that both participate, or both do not participate. The evolutionary stable strategy (ESS) of the system is E_1_ (0,0) and E_4_ (1,1). When the initial state is in the E_1_E_2_E_3_E_5_ field (set as field I), the system tends to the equilibrium point E_1_ (0,0), that is, both will not participate; when the initial state is in the E_2_E_3_E_4_E_5_ field (set as field II), the system will converge to the equilibrium point E_4_ (1,1), that is, both will participate. The evolutionary phase diagram is shown in Figure 2.

### 3.4. The Analysis of the Impact of Factors

Combining the above analysis results, the present study focuses on Scenario 9, that is, under the condition of mixed strategy Nash equilibrium, two stable strategies exist for the evolutionary game of the contractors and the manufacturers as {participation, participation} and {non-participation, non-participation}. Participating in the development of the CDW recycling industry is the Pareto optimal results of the game, but the two strategies are stable, and the possibility of the evolutionary result in which direction is determined by the square of the filed I and the filed II. When S_I_ = S_II_, the probability of the two players choosing the strategy is the same; when S_I_ > S_II_, the probability of both non-participating is greater than the probability of both participating; when S_I_ < S_II_, the probability that both players choose to participate is greater than the probability that they will not participate. Currently, the environmental and resources problems are increasingly serious, so we anticipate that both players will participate in the CDW recycling industry. Therefore, we need to increase the S_II_ and reduce the S_I_. By analyzing the factors affecting S_I_, it can be transformed into an analysis of the factors that influence the choice of participation strategy. The direction of the factors affecting S_I_ is opposite to the direction of the participation of both players.

(10) $$S_{1}=\frac{x^{*}\cdot y^{*}}{2}=\frac{\left(R+(1-\alpha )C_{2}-{\left(K_{1}C_{1}\right)}^{a_{2}}+C_{1})\cdot (-\lambda_{1}(\theta T{)}^{b_{1}}-(K_{2}C_{3}{)}^{a_{1}}+C_{3}\right)}{2\cdot \left[R+(\beta F{)}^{a_{2}}\right]\cdot (\gamma F{)}^{a_{1}}}\;$$

According to Equation (10), there are 16 factors influencing the evolution of the system, and further conclusions can be drawn, as shown in Table 4.

Conclusion 1: The higher the income *R* and the cost of producing finished products *C*_2_ of the manufacturers, the greater the probability that both will not participate. When the production cost proportional coefficient *α*, the recycling cost *C*_1_, and the cost of producing finished products *C*_2_ are constant, the increase in the income *R* will make marginal subsidies and benefits obtained after recycling which are negligible compared to the income, so the manufacturers will choose not to participate.

Conclusion 2: The higher the factors, including the production cost proportional coefficient *α*; the benefits of recycling *F*; the internalization degree of the two stakeholders to these effects *β*, *γ*; the government subsidy coefficient *K*_1_, *K*_2_; the probability of discovering the illegal dumping *θ*; the *T* of fine for dumping CDW; and the loss aversion coefficient *λ*_1_ of the contractors, the greater the probability of both participating.

Conclusion 3: The effects of the marginal decline degrees a, b of the contractors and manufacturers on the system evolution are affected indirectly by other factors. Therefore, these effects are uncertain.

Conclusion 4: When the recycling cost *C*_1_ is less than a certain value *Z*_1_, the probability of participation of both players is large. When the recycling cost *C*_1_ is greater than a certain value *Z*_1_, the probability of participation of both players is small. The specific value is $Z_{1}=\sqrt[{1-a_{2}}]{a_{2}K_{1}}/K_{1}$. It is related to the government subsidy coefficient *K*_1_ for the manufacturers and the degree of marginal decline of the value of the gain a_2_.

Conclusion 5: When the sorting cost *C*_3_ is less than a certain value *Z*_2_, the probability of participation of both players is large. When the recycling cost *C*_3_ is greater than a certain value *Z*_2_, the probability of participation of both players is small. The specific value is $Z_{2}=\sqrt[{1-a_{1}}]{a_{1}K_{2}}/K_{2}$. It is related to the government subsidy coefficient *K*_2_ for the contractors and the degree of marginal decline of the value of the gain a_1_.

In summary, in the process of the development of the CDW recycling industry, the probability of joint participation of manufacturers and contractors is affected by the production cost, technology, subsidies, recycling benefits, and the degree of perception of stakeholders. However, the evolutionary path and final result of both stakeholders’ behavioral decision-making are also affected by the choice of the initial strategy. In the following, the effects of various factors on the recycling behavior of the stakeholders will be graphically demonstrated through numerical simulation.

## 4. Numerical Simulation

Based on the above assumptions and analysis, considering the probability of recycling for contractors and manufacturers, in order to explore the impact of the initial strategy and different factors on the choice of CDW behavior more intuitively, we use Matlab to conduct simulation analysis of the evolutionary game to observe changes in the evolutionary state of strategic choices for contractors and manufacturers, along with changes in the initial strategy and factor values.

In view of the individual differences in the construction industry and the variety of CDW types, the setting of experimental parameters in this paper satisfies the participation constraints: ${\left\{ \begin{array}{l} (K_{1}C_{1}{)}^{a_{2}}-C_{1}-C_{2}<R-\alpha C_{2}<R+{\left(\beta F\right)}^{a_{2}}+(K_{1}C_{1}{)}^{a_{2}}-C_{1}-C_{2}\\ (K_{2}C_{3}{)}^{a_{1}}-C_{3}<-\lambda_{1}{\left(\theta T\right)}^{b_{1}}<(\gamma F{)}^{a_{1}}+(K_{2}C_{3}{)}^{a_{1}}-C_{3} \end{array}\right.}_{}$.

The parameter values are as shown in Table 5.

### 4.1. The Impact of Initial Strategy Differences on Evolutionary Results

**Figure 3:**
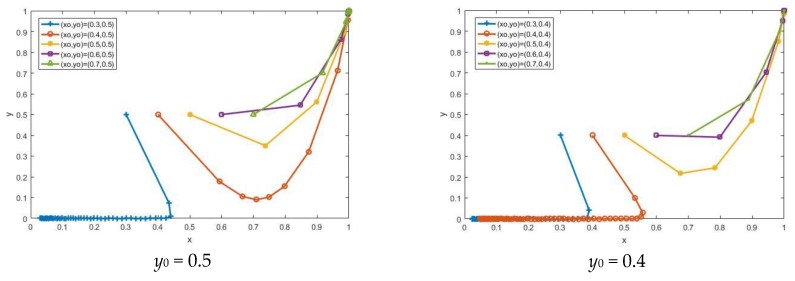
The impact of changes in *x*_0_ on the evolutionary result.

**Figure 4:**
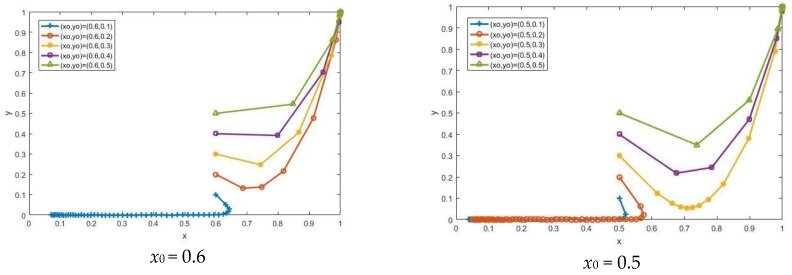
The impact of changes in *y*_0_ on the evolutionary result.

With other parameters unchanged, we change the value of the initial strategy (*x*_0_,*y*_0_) to study the impact of the difference on the evolutionary results. When *y*_0_ = 0.5 is fixed, *x*_0_ increases from 0.3, 0.4, 0.5, 0.6, and 0.7 in sequence. When *x*_0_ = 0.4, the system changes from the non-participation state to the participation state. When *y*_0_ = 0.4 is fixed, *x*_0_ increases sequentially. When *x*_0_ = 0.5, the system state changes. As *x*_0_ continues to increase, its evolutionary rate increases, as shown in Figure 3. When *x*_0_ = 0.6 is fixed, *y*_0_ increases from 0.1, 0.2, 0.3, 0.4, and 0.5 in sequence. When *y*_0_ is 0.2, the system changes from the non-participation state to the participation state. When *x*_0_ = 0.5 is fixed, *y*_0_ increases sequentially, and when *y*_0_ is 0.3, the system state changes. As *y*_0_ continues to increase, its evolutionary rate increases, as shown in Figure 4. The high degree of participation of any stakeholder in the CDW recycling industry will cause the system to transit to the participation state more quickly, which shows that the behavioral decision-making on recycling between stakeholders is mutually influenced.

### 4.2. The Impact of Factors on Evolutionary Results

**Figure 5:**
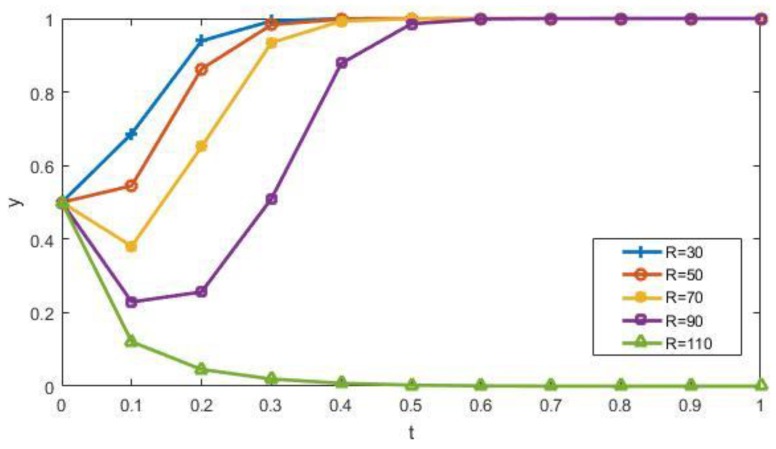
The impact of *R* on the evolutionary results.

With other factors being constant, we study the impact of *R* = 30, 50, 70, 90, 110, respectively, on the evolutionary results of the manufacturers’ strategy, as shown in Figure 5. With the increase of income *R*, the rate of the manufacturers tending to participate in recycling will slow down. Especially, when *R* = 70, 90, the proportion of the manufacturers that choose participation (*y*) decreases at the beginning. Based on the nature of the evolutionary game, the strategy of the manufacturers is adjusted over time by the interaction of other factors, such as the subsidies and benefits obtained after recycling. Thus, the manufacturers will eventually choose to participate. However, when *R* is high enough, such as *R* = 110, it makes marginal subsidies and benefits obtained after recycling negligible compared with the income, so the manufacturers will choose raw material to produce products. To a certain extent, it verifies the rationality of Conclusion 1.

**Figure 6:**
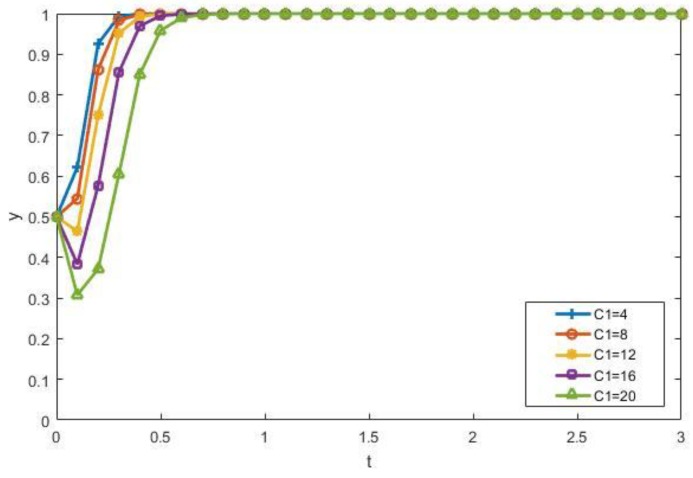
The impact of *C*_1_ on the evolutionary results.

**Figure 7:**
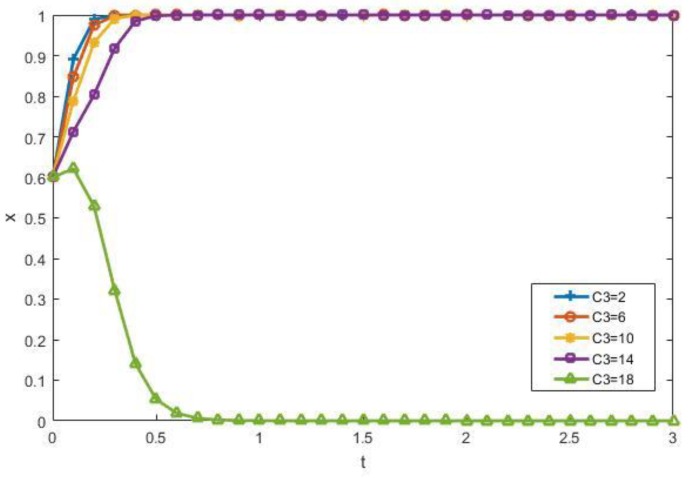
The impact of *C*_3_ on the evolutionary results.

The impact of recycling costs *C*_1_ and the sorting cost *C*_3_ on the evolutionary results of manufacturers and contractors is discussed as follows. Figure 6 shows that when other factors are constant, the increase in *C*_1_ inhibits the enthusiasm of manufacturers to participate. Figure 7 shows that when other factors do not change, the increase of *C*_3_ inhibits the enthusiasm of the contractors to choose to participate in the recycling process. What is more, when *C*_3_ exceeds a certain value, the contractors would rather dump illegally instead of participating in the recycling of CDW.

**Figure 8:**
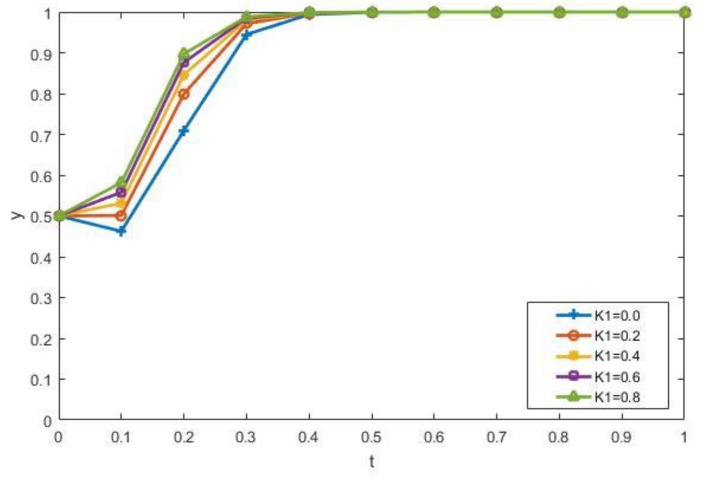
The impact of *K*_1_ on the evolutionary results.

**Figure 9:**
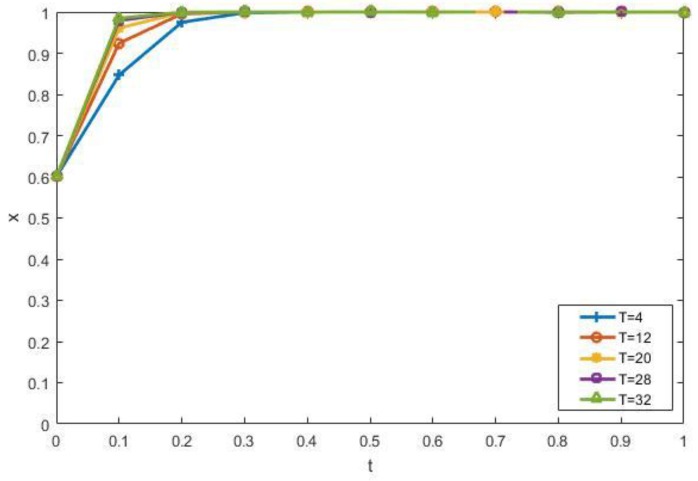
The impact of *T* on the evolutionary results.

We discuss the impact of government environmental regulations on the evolutionary results. Figure 8 reveals the impact of changes in the government subsidy coefficient *K*_1_ of manufacturers on strategies. As can be seen from the Figure 8, the higher *K*_1_ is, the more it tends to choose recycling production. In addition, when the *K*_1_ values are 0.4, 0.6, and 0.8, the evolutionary curve almost coincides. Therefore, the government can select the optimal coefficient when determining the subsidy coefficient. The impact of changes in *K*_2_ is similar to *K*_1_, and is not repeated here. When other factors remain unchanged, the higher the fine for illegal dumping of the contractors, the more the contractors will be forced to choose the recycling of CDW, as shown in Figure 9. When *T* = 12, 20, and 28, the evolution curves almost coincide. Therefore, the government does not have to formulate severe punishments to guide the contractors to participate in the recycling of CDW. These all verify the rationality of Conclusion 2.

The impact of self-perceived gain, diminishing marginal degree of loss and the loss avoidance coefficient is discussed. Take a_2_, *λ*_1_ as an example. In view of the numerical simulation data, the following inequalities are satisfied.

$$\frac{\partial S_{1}}{\partial a_{2}}=\frac{-{\left(\beta F\right)}^{a_{2}}\cdot {\left(K_{1}C_{1}\right)}^{a_{2}}\cdot \ln\;K_{1}C_{1}-{\left(\beta F\right)}^{a_{2}}\cdot \left[R+(1-\alpha )C_{2}-{\left(K_{1}C_{1}\right)}^{a_{2}}+C_{1}\right]\cdot \ln\;\beta F}{2\cdot {\left(\gamma F\right)}^{a_{1}}\cdot {\left[R+{\left(\beta F\right)}^{a_{2}}\right]}^{2}}<0\;$$

**Figure 10:**
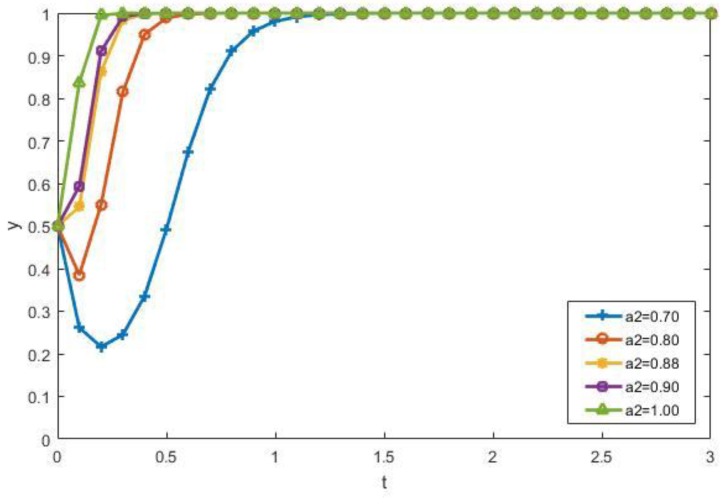
The impact of a_2_ on the evolutionary results.

**Figure 11:**
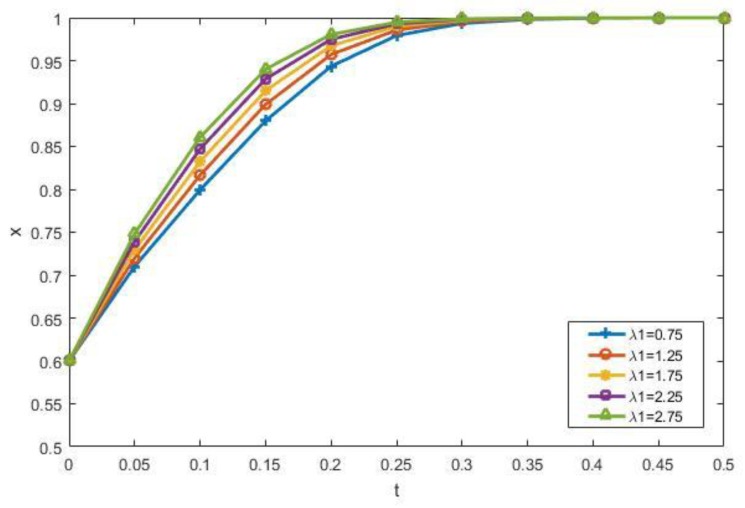
The impact of *λ*_1_ on the evolutionary results.

Therefore, it can be clearly seen from Figure 10 that when a_2_ is low (a_2_ = 0.7 or a_2_ = 0.8), which means that the gains of participating perceived by the manufacturers are low, the manufacturers initially tend not to participate. So the proportion of the manufacturers that choose participation (*y*) decreases at first. The strategy of the manufacturers is adjusted over time by the interaction of other factors. Thus, the manufacturers will eventually choose to participate. We can also find that with the increase of a_2_, that is, the better the perceived benefit of the manufacturers, the faster it will convert to the participation state. When increasing the contractors’ loss-avoidance coefficient *λ*_1_, which indicates that they believe the punishment will become more and more serious when the government finds their illegal dumping of CDW, they will eventually choose to participate in the recycling of CDW, as shown in Figure 11.

## 5. Conclusions

This paper explores the internal mechanism of the behavioral decision-making of CDW recycling under environmental regulation by combining prospect theory with evolutionary game theory. The uncertain gains and losses in the evolutionary game’s payoff matrix are replaced by the perceived utility functions of the prospect theory, which further embodies the limited rationality of stakeholders from perception to behavioral decision-making, making the game’s conclusions closer to the behavioral decision-making of stakeholders involved in the recycling of CDW in reality. Through the analysis of the evolutionary game model, including contractors and manufacturers of construction materials, the conclusions and further research directions are drawn as follows.

When the perceived benefits for participation of one or both stakeholders, including the contractors and the manufacturers of construction materials under the environmental regulation, exceed those for non-participation, the CDW recycling system will eventually evolve to a stable state in which both stakeholders choose to participate in the strategy, as described in Scenario 1,6,7.

The contractors should take the social responsibility in line with the principle “the producer should be held accountable”. While resorting to self-compliance, the contractors must strengthen CDW management and reduce the *C*_3_ cost of sorting_._ The manufacturers of construction materials should also undertake social responsibilities, introduce technology, reduce the *C*_2_ cost of producing finished products as much as possible, and control the cost of disposal of CDW near ${Ｃ}_{1}=\sqrt[{1-a_{2}}]{a_{2}K_{1}}/K_{1}$, so as to increase the recycling rate of CDW.

The increase of the parameters including the government’s subsidy coefficients *K*_1_ and *K*_2_ for contractors and manufacturers of construction materials; the probability of discovering the illegal dumping *θ*; the fine *T*; the environmental and social effects *F*; and the internalization degree of the two stakeholders to these effects *β*, *γ*, has a promoting effect on the evolution of the system. The government can refer to the model proposed in this study and take corresponding measures to formulate the best environmental regulation policy based on other parameters in the context where the recycling effects benefit the society.

The effects of the marginal decline degrees a, b of the contractors and manufacturers of construction materials on the system evolution are affected indirectly by other factors; therefore, those effects are uncertain. However, the government should strengthen the recycling of CDW and publicize environmental regulation policies to increase the perceived benefits of stakeholders and reduce the perceived losses of stakeholders, thereby promoting the final evolution of the system to a stable state in which both stakeholders choose to participate in the strategy.

This research, based on the perspective of bounds rationality, extended the evolutionary game theory with prospect theory to study the internal mechanism of the behavioral decision-making of contractors and manufacturers of construction materials and demonstrate the impact of key factors such as the choice of the initial strategy, production cost, technology, subsidies, recycling benefits, and the degree of perception of stakeholders on the decision-making process, so as to develop new ideas for promoting the development of the CDW recycling industry, thereby providing a theoretical basis for the government to formulate environmental regulatory policies.

The present work could be extended in various ways, some of which we suggest here. There is a certain deviation between the assumptions and reality in this paper. For example, we assume that the same income results from recycled and raw materials to study the impact of environmental regulation, recycling benefits, and production cost on the behavioral decision-making of manufacturers, which ignores the volatility of the market. It is meaningful to study the impact of price incentives on behavioral decision-making. Moreover, in this study, the environmental regulations are static and the stakeholders considered are only the contractors and the manufacturers. However, the environment of regulation which the CDW recycling industry is faced with is constantly changing and its sustainable development also involves many other stakeholders, such as recycling processing centers and the vast majority of consumers in the real estate market. How to establish a multivariate evolutionary game model under dynamic environment regulation; analyze its operation mechanism, behavioral decision-making, and stable state; as well as propose systematic development countermeasures, are the topics that we will study and discuss in the future. Finally, in this study, manufacturers and contractors are independent of each other, and their behavioral decision-making is based on their own interests. Thus, a potential extension is to study the contract between the contractors and the manufacturers to promote the development of the recycling of CDW.

## Figures and Tables

**Table 1:** The perceived payoff matrix between contractors and manufacturers.

		**Manufacturers**	
		Participation (*y*)	Non-Participation (1 − *y*)
**Contractors**	Participation (*x*)	$\begin{array}{l} V_{1}(\gamma F)+V_{1}(K_{2}C_{3})-C_{3},\\ R-C_{1}-C_{2}+V_{2}(\beta F)+V_{2}(K_{1}C_{1}) \end{array}$	$\begin{array}{l} V_{1}(K_{2}C_{3})-C_{3},\\ \;R-\alpha C_{2}\; \end{array}$
	Non-participation (1 − *x*)	$\begin{array}{c} V_{1}(-\theta T),\\ V_{2}(K_{1}C_{1})-C_{1}-C_{2} \end{array}$	$\begin{array}{l} V_{1}(-\theta T),\\ R-\alpha C_{2} \end{array}$

**Table 2:** The formula of the determinant for each equilibrium point.

Equilibrium Point	Det J
E_1_ (0,0)	$\left[V_{1}(K_{2}C_{3})-C_{3}-V_{1}(-\theta T)\right]\cdot \left[V_{2}(K_{1}C_{1})-C_{1}-C_{2}-(R-\alpha C_{2})\right]$
E_2_ (1,0)	$\left[C_{3}+V_{1}(-\theta T)-V_{1}(K_{2}C_{3})\right]\cdot \left[V_{2}(\beta F)+V_{2}(K_{1}C_{1})-C_{1}-(1-\alpha )C_{2})\right]$
E_3_ (0,1)	$\left[V_{1}(\gamma F)+V_{1}(K_{2}C_{3})-C_{3}-V_{1}(-\theta T)\right]\cdot \left[R+(1-\alpha )C_{2}-V_{2}(K_{1}C_{1})+C_{1}\right]$
E_4_ (1,1)	$\left[V_{1}(-\theta T)-V_{1}(\gamma F)-V_{1}(K_{2}C_{3})+C_{3}\right]\cdot \left[\left(1-\alpha )C_{2}-V_{2}(K_{1}C_{1})+C_{1}-V_{2}(\beta F\right)\right]$
E_5_ (x*,y*)	$\begin{array}{l} \frac{-\left[V_{1}(\gamma F)-V_{1}(-\theta T)+V_{1}(K_{2}C_{3})-C_{3}\right]\cdot \left[V_{1}(-\theta T)-V_{1}(K_{2}C_{3}+C_{3})\right]}{V_{1}(\gamma F)}\\ {}*\frac{\left[V_{2}(\beta F)+V_{2}(K_{1}C_{1})-C_{1}-(1-\alpha )C_{2}\right]\cdot \left[R+(1-\alpha )C_{2}-V_{2}(K_{1}C_{1})+C_{1}\right]}{R+V_{2}(\beta F)} \end{array}$

**Table 3:** The formula of the trace for each equilibrium point.

Equilibrium Point	Tr J
E_1_ (0,0)	$\left[V_{1}(K_{2}C_{3})-C_{3}-V_{1}(-\theta T)\right]+\left[V_{2}(K_{1}C_{1})-C_{1}-C_{2}-(R-\alpha C_{2})\right]$
E_2_ (1,0)	$\left[C_{3}+V_{1}(-\theta T)-V_{1}(K_{2}C_{3})\right]+\left[V_{2}(\beta F)+V_{2}(K_{1}C_{1})-C_{1}-(1-\alpha )C_{2})\right]$
E_3_ (0,1)	$\left[V_{1}(\gamma F)+V_{1}(K_{2}C_{3})-C_{3}-V_{1}(-\theta T)\right]+\left[R+(1-\alpha )C_{2}-V_{2}(K_{1}C_{1})+C_{1}\right]$
E_4_ (1,1)	$\left[V_{1}(-\theta T)-V_{1}(\gamma F)-V_{1}(K_{2}C_{3})+C_{3}\right]+\left[\left(1-\alpha )C_{2}-V_{2}(K_{1}C_{1})+C_{1}-V_{2}(\beta F\right)\right]$
E_5_ (x*,y*)	0

**Table 4:** The impact of factor change on the system evolution result.

Factor	*R* ↑	*C*_2_ ↑	*α* ↑	*β* ↑	*γ* ↑	*F* ↑	*K*_1_ ↑	*K*_2_ ↑	*θ* ↑	*T* ↑	*λ*_1_ ↑	a_1_ ↑	a_2_ ↑	b_1_ ↑	*C*_1_ ↑	*C*_3_ ↑
S_I_	↑	↑	↓	↓	↓	↓	↓	↓	↓	↓	↓	U ^1^	U ^1^	U ^1^	↘↗	↘↗
S_II_	↓	↓	↑	↑	↑	↑	↑	↑	↑	↑	↑	U ^1^	U ^1^	U ^1^	↗↘	↗↘

^1^ The impact of factors on the system is uncertain.

**Table 5:** The parameter values.

(*x*_0_,*y*_0_)	*R*	*α*	*C*_1_	*C*_2_	*C*_3_	*T*	*θ*	*β*	*γ*	*F*	*K*_1_	*K*_2_	*λ*_1_	a_1_	a_2_	b_1_
(0.6,0.5)	50	0.9	8	12	4	4	0.5	0.3	0.3	150	0.4	0.2	2.25 ^1^	0.88 ^1^	0.88 ^1^	0.88 ^1^

^1^ The parameters are based on the study of Tversky et al. [31].
